# Influence of Rho/ROCK inhibitor Y-27632 on proliferation of equine mesenchymal stromal cells

**DOI:** 10.3389/fvets.2023.1154987

**Published:** 2023-06-06

**Authors:** Michaela Melzer, Janina Burk, Deborah J. Guest, Jayesh Dudhia

**Affiliations:** ^1^Equine Clinic (Surgery, Orthopedics), Faculty of Veterinary Medicine, Justus Liebig University, Giessen, Germany; ^2^Department of Clinical Sciences and Services, Royal Veterinary College, Hertfordshire, United Kingdom

**Keywords:** Y-27632, mesenchymal stem cell, equine, senescence, Rho/ROCK

## Abstract

Mesenchymal stromal cells (MSC) isolated form bone marrow and adipose tissue are the most common cells used for cell therapy of orthopedic diseases. MSC derived from different tissues show differences in terms of their proliferation, differentiation potential and viability in prolonged cell culture. This suggests that there may be subtle differences in intracellular signaling pathways that modulate these cellular characteristics. The Rho/ROCK signaling pathway is essential for many cellular functions. Targeting of this pathway by the ROCK inhibitor Y-27632 has been shown to be beneficial for cell viability and proliferation of different cell types. The aim of this study was to investigate the effects of Rho/ROCK inhibition on equine MSC proliferation using bone marrow-derived MSC (BMSC) and adipose-derived MSC (ASC). Primary ASC and BMSC were stimulated with or without 10 ng/mL TGF-β3 or 10 μM Y-27632, as well as both in combination. Etoposide at 10 μM was used as a positive control for inhibition of cell proliferation. After 48 h of stimulation, cell morphology, proliferation activity and gene expression of cell senescence markers p53 and p21 were assessed. ASC showed a trend for higher basal proliferation than BMSC, which was sustained following stimulation with TGF-β3. This included a higher proliferation with TGF-β3 stimulation compared to Y-27632 stimulation (*p* < 0.01), but not significantly different to the no treatment control when used in combination. Expression of p21 and p53 was not altered by stimulation with TGF-β3 and/or Y-27632 in either cell type. In summary, the Rho/ROCK inhibitor Y-27632 had no effect on proliferation activity and did not induce cell senescence in equine ASC and BMSC.

## Introduction

Mesenchymal stromal cells (MSC) are a promising therapeutic tool for the treatment of orthopedic diseases (1). MSC can be derived in relatively high numbers from various tissues, such as blood, fat, bone marrow or umbilical cord blood (2–6). The most commonly investigated and used are bone marrow-derived MSC (BMSC). However, since the collection and cultivation of BMSC is associated with some limitations, such as painful aspiration technique of bone marrow, low cell yield, and early cell aging (7), adipose-derived MSC (ASC) have appeared to be a good alternative. While ASC and BMSC are comparable in terms of their morphology and cell surface markers, they show several differences regarding their differentiation and proliferation ability (8–10). This suggests that there may exist subtle differences in intracellular pathways that modulate these cellular characteristics.

The Rho/Rho-associated protein kinase (Rho/ROCK) signaling pathway plays a critical role in the regulation of many cellular functions. One of the major targets of the Rho/ROCK signaling pathway is the regulation of phosphorylation of myosin-light-chain phosphatase and a number of other phosphokinases and cytoskeleton-binding proteins (11). Through this, Rho/ROCK controls cytoskeletal contraction which is essential for many basic cellular processes, including apoptosis, migration, proliferation, and differentiation (11). Thus, the Rho/ROCK pathway is often targeted to influence cell proliferation and differentiation. For this purpose, there are several small molecule inhibitors of ROCK which affect cells differently depending on the cell type and combinations of growth factors. For example, the competitive ROCK inhibitor Y-27632 can inhibit differentiation triggered by extracellular matrix and mechanical stimuli (12–14) but can promote differentiation triggered by paracrine factors (15–17).

Inhibition of ROCK however has variable effects on other cellular functions. While complete silencing of ROCK protein via gene knockout or potent Rho/ROCK inhibitors promotes cellular senescence and limits proliferation (18), Y-27632 appears to be beneficial for cell viability and proliferation. It can promote long-term proliferation of human embryonic stem cell (ESC)-derived endothelial cells and primary keratinocytes and reduces cell senescence (19–21). Similarly, human ESC show improved viability, cell growth and regeneration ability after cryopreservation when supplemented with Y-27632 (22, 23).

Thus, the modulation of the Rho/ROCK signaling pathway with Y-27632 has proven to be a simple, efficient and versatile approach in embryonic stem cell applications for regenerative medicine research. Should these desirable properties of the Y-27632 inhibitor be applicable to MSC, with the potential positive influence on differentiation and proliferation, the inhibitor would represent a promising candidate for preconditioning of MSC during cell expansion. However, there are no studies to date on the effect of Y-27632 on equine MSC proliferation. Therefore, the aim of the study was to investigate the effects of Rho/ROCK inhibition on the proliferation of equine MSC, taking different tissues of origin into account by comparing ASC with BMSC.

## Methods

### Cell culture and treatment

Cell culture ingredients were purchased from ThermoFisher Scientific (Warrington, United Kingdom) unless stated otherwise. Adipose-derived MSC and bone marrow-derived MSC were collected from eight different donors (ASC *n* = 4, BMSC *n* = 4). The use of equine MSC was approved by the Royal Veterinary College Clinical Research Ethical Review Board (URN 2022 2127-2 and URN 2021 2035-2). The donors were warmbloods, warmblood crosses (BMSC, age 6–15 years) or welsh cob ponies (ASC, age 2–5 years).

MSC from bone marrow aspirates were prepared as previously described using a standardized protocol in our laboratory for use in the equine clinic (24). Briefly, 10 mL of bone marrow aspirate was diluted with an equal volume of Dulbecco’s PBS and layered over 15 mL of Lymphoprep (Stem Cell Technologies, Cambridge, United Kingdom) and centrifuged at 1,200 RCF for 10 min. The buffy layer containing the mononuclear cell fraction was removed and the cells seeded in tissue culture flasks (T-75, Falcon) in cell culture media (DMEM, 1 g/L glucose; Gibco^®^, 0.11 mg/mL sodium pyruvate) supplemented with 10% FCS (Gibco^®^) and 1% Penicillin–Streptomycin (Gibco^®^). Plastic adherent MSC were expanded and passaged to passage number 2 or 3 with an estimated population doubling level of 11–12. Cells were resuspended in cell freezing medium (Cellbanker 2, AMS Biotech, United Kingdom) and stored frozen in liquid nitrogen until used for experiments. Further characterization of the BMSC was not performed because we have previously characterized MSC prepared by this standardized protocol for surface markers and trilineage differentiation (24) and a position statement by Guest et al. recommends that it is not necessary to characterize every batch that utilizes a standard protocol (25).

For the isolation of ASC, 15 g adipose tissue of the dorsal gluteal muscle were collected in same medium as BMSC. The tissue was washed, diced and incubated with 1 mg/mL collagenase I for 1 h at 37°C. After digestion, cells were recovered by centrifugation at 350 RCF for 10 min and then washed two times following resuspension in cell culture media. Cells were seeded into a 10 cm dish in cell culture media at 1,000–5,000 cells cm^2^. Plastic adherent MSC were expanded, passaged and stored frozen in liquid nitrogen until used for experiments. ASCs were characterized for trilineage differentiation and expression of CD90, CD29, CD44, CD14 (neg) and CD79a (neg) using assays previously described (26).

For experiments, aliquots of cells were rapidly thawed and cultured in cell culture medium at a seeding density of 5,000 cells/cm^2^. Cells were allowed to attach and recover for 24 h before stimulating with 10 ng/mL TGF-β3 (R&D Systems^®^, Abingdon, United Kingdom), 10 μM Y-27632 (Tocris, Bioscience, Bistrol, United Kingdom), both in combination or with 10 μM etoposide (ab120227, Abcam, Cambridge, United Kingdom) which was used as positive control to induce senescence (27). Concentrations of TGF-β3 and Y-27632 were chosen based on previous studies [(17, 28), respectively]. For the combined treatment, cells were preincubated with Y-27632 for 2 h before adding TGF-β3. Assessments were performed 48 h after stimulation.

### Proliferation assay

Cell proliferation was assayed by EdU labeling with the Click-iT^®^ Plus EdU Imaging Kit (ThermoFisher Scientific, Warrington, United Kingdom) according to manufacturer’s instructions. Briefly, cells were incubated with 10 μM EdU for 2 h, fixed with 3.7% formaldehyde for 15 min and stained with the Click-iT^®^ reaction cocktail. Cells were then stained with anti-Ki-67 antibody (ab281847, Abcam, Cambridge, United Kingdom) for 2 h. Cell were counterstained with DAPI and then imaged with fluorescence microscopy (EVOS^®^ FL Imaging System, ThermoFisher Scientific, Warrington, United Kingdom). Images were quantified for cell numbers with ImageJ (Version 1.53 Fiji). A minimum of 500 cells per stimulation group were counted for positive staining of each label. A ratio of EdU or Ki-67 positive cells to total cells (DAPI positive) was calculated.

### Gene expression analysis

Gene expression of the senescence markers p21 and p53 (29) were analyzed by real-time PCR. GAPDH was used as a reference gene. Total RNA of equine cells was isolated using the RNeasy Mini Kit (Qiagen, United Kingdom) with additional DNase digestion (Qiagen) according to manufacturer’s instructions. RNA was then converted to cDNA using the Reverse Transcriptase RevertAidH Minus kit (ThermoFisher Scientific). 50 ng cDNA was mixed with primers (Table 1) and QuantiNova™ SYBR^®^ Green PCR kit to perform real-time PCR using the CFX96™ Real Time System (Bio-Rad, Hercules, United States). For relative quantification, gene expression ratios and fold changes were calculated with the Pfaffl method (30) and normalized to day control.

**Table 1:** Equine primers.

Gene	Primer sequence	Gene	Product length (bp)
p21	FOR: ACATACTCTGCTTGCCACCC REV: GGCCCCCTTCAAAGTGCTAT	XM_023624844.1	332
p53	FOR: ACTCCAGCCACCTGAAGTCT REV: GGGGACAGGAAGCAGAGAAT	XM_023651624.1	110
GAPDH	FOR: CATCAAATGGGGCGATGCTG REV: TGCACTGTGGTCATGAGTCC	NM_001163856.1	285

### Statistical analysis

Statistical analysis was performed using IBM SPSS Statistics 28 software (IBM Deutschland GmbH, Ehningen, Germany). As data were not normally distributed, non-parametric Friedman tests with Bonferroni-adjusted post-hoc tests were used. Differences were considered significant at *p* ≤ 0.05. Graphs were designed with GraphPad Prism 9.4.1 (GraphPad Software, San Diego, United States).

## Results

**Figure 1:**
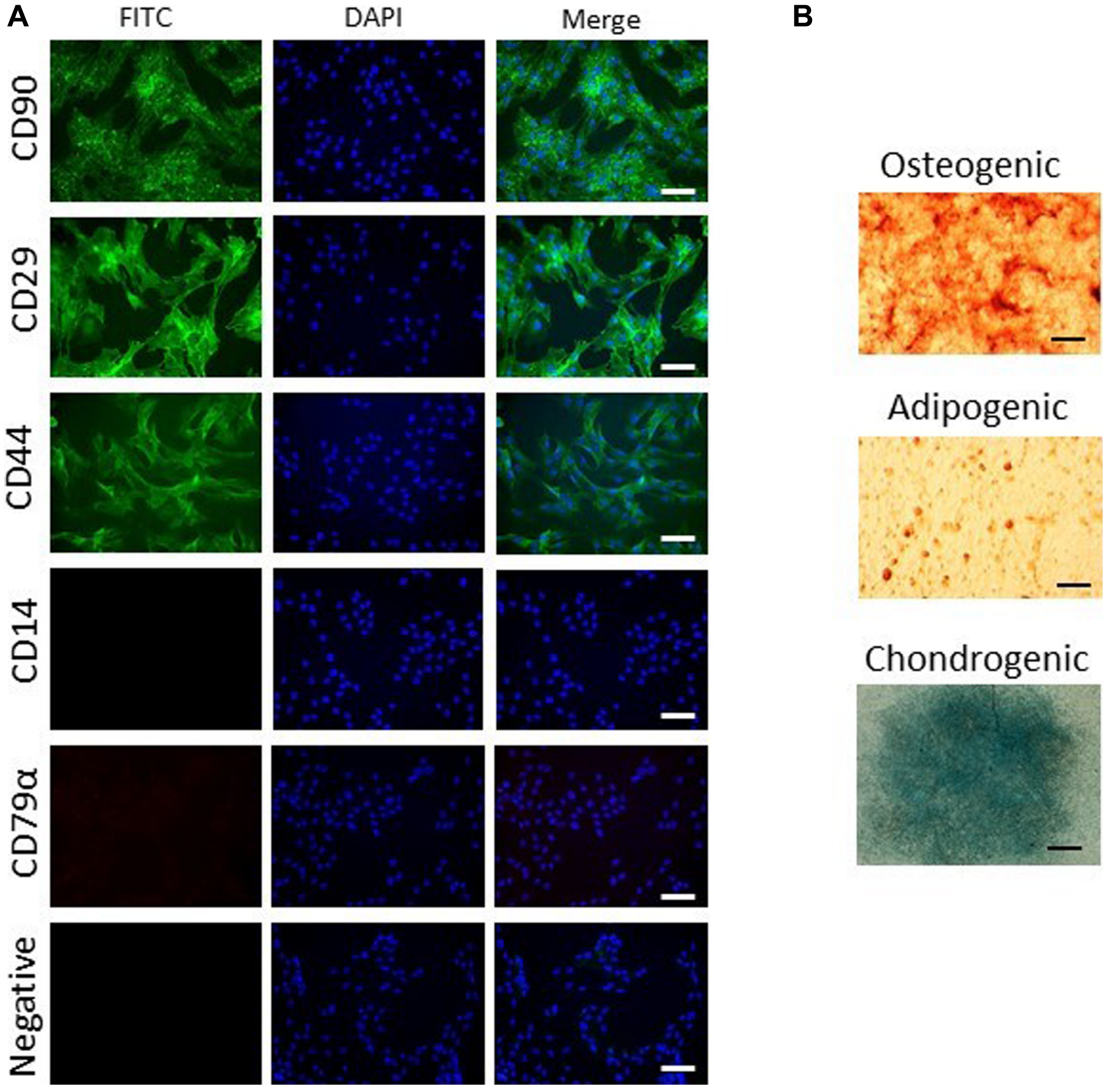
Characterization of ASCs. Representative images of three biological replicates show marker expression **(A)** and trilineage differentiation **(B)**. ASCs express CD90, CD29, and CD44 but not CD14 or CD79α (FITC, scale bar = 50 μm). ASCs undergo trilineage differentiation into cartilage (alcian blue staining, scale bar = 1 mm), bone (alizarin red staining, scale bar = 50 μm) and fat (oil red O staining, scale bar = 50 μm).

**Figure 2:**
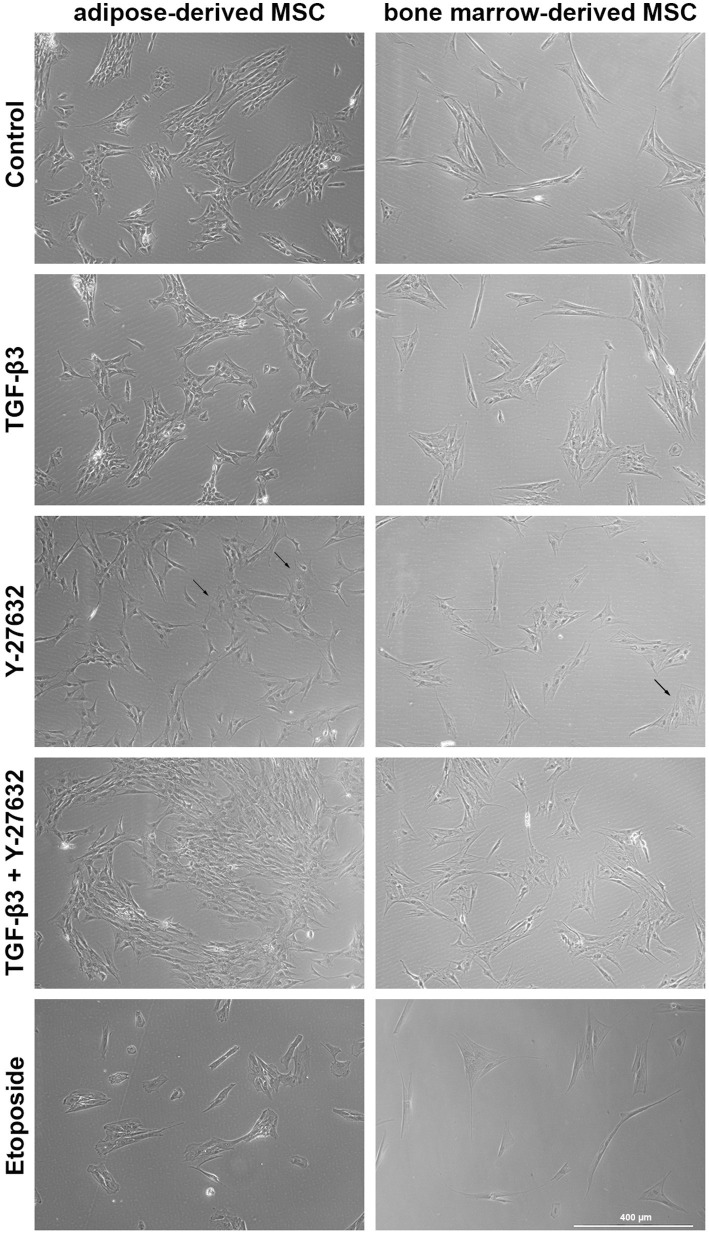
Brightfield microscopy of ASC and BMSC. Cells were treated with 10 ng/mL TGF-β3 or 10 μM Y-27632, and in combination or with 10 μM etoposide and analyzed after 48 h incubation. Representative images show cell morphology and confluency after stimulation. Arrows highlight single flattened cells with poor contrast.

ASC were capable of differentiation into adipocytes, osteoblasts and chondrocytes. ASC were positive for CD90, CD29, and CD44 and negative for CD14 and CD79α (Figure 1). ASC and BMSC showed a spindle-shaped, fibroblast-like morphology in cell culture medium, with BMSC appearing more elongated than ASC as assessed by microscopy (Figure 2). BMSC were slower in reaching confluency than ASCs.

In the presence of TGF-β3 alone or with Y-27632, BMSC exhibited a change in morphology which was more marked than in ASC. ROCK inhibition with Y-27632 resulted in the loss of the elongated spindle shape and into a broader rectangular cell shape in both cell types. In addition, some cells had a flattened circular appearance with poor contrast in phase contrast microscopy. These morphological changes were less marked with TGF-β3. The addition of etoposide induced a rounded cell shape and arrested cell proliferation, as indicated by reduced cell confluency.

**Figure 3:**
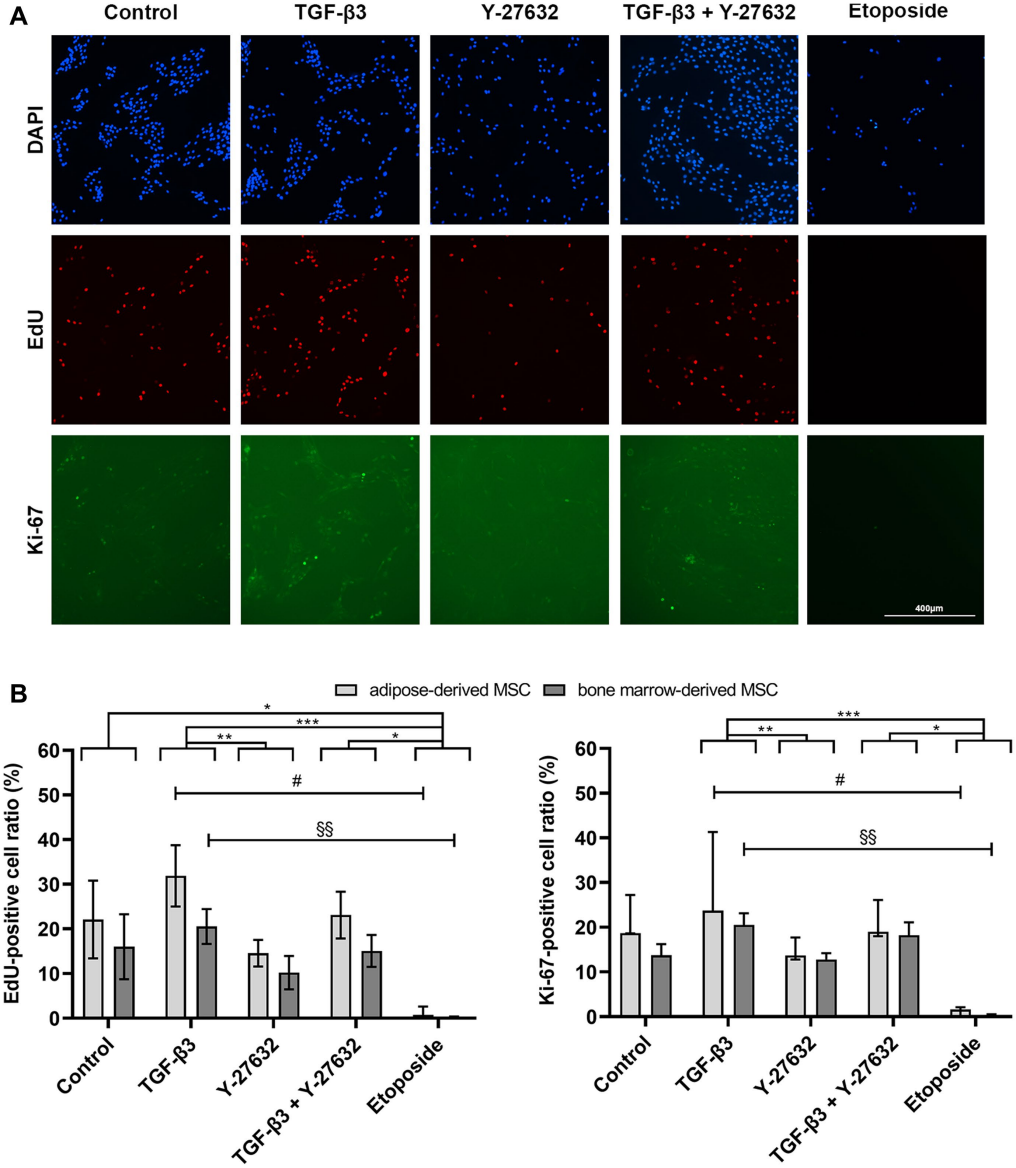
Fluorescence images of EdU- and Ki-67-labeling. ASC and BMSC were treated with 10 ng/mL TGF-β3 or 10 μM Y-27632, and in combination, or with 10 μM etoposide and analyzed after 48 h incubation. Representative images of ASC show nuclei staining (DAPI) and EdU- and Ki-67-labeling **(A)**. The diagrams represent quantitative image analysis results of proliferation activity of ASC and BMSC treatment groups **(B)**. Bars represent the median values, error bars the 95% confidence intervals. The asterisks indicate significant differences between the corresponding groups when the data of ASC and BMSC are combined (* corresponds to *p* < 0.05; ** corresponds to *p* < 0.01; *** corresponds to *p* < 0.001; *n* = 7). The hashtag and paragraph symbols mark differences between the indicated groups for ASC and BMSC alone (#: ASC; *n* = 3; §: BMSC; *n* = 4).

Consistent with this, both EdU and Ki-67 labeling was almost completely inhibited by etoposide stimulation in both BMSC and ASC (EdU and Ki-67 label: *p* < 0.05 compared to control, TGF-β3 + Y-27632, and TGF-β3; data of BMSC and ASC combined; Figure 3). Although there was a trend for an increase in proliferation with TGF-β3 compared to controls, this was not significant for BMSC or for ASC. There was a small decrease in proliferation with Y-27632, but was not significant compared to the control. There was a significant difference in proliferation between TGF-β3 and Y-27632 (EdU label: *p* < 0.01 for TGF-β3 compared to Y-27632; data of BMSC and ASC combined). When both compounds were used in combination, proliferation recovered to control levels (no significant difference). Proliferation of ASC and BMSC in the etoposide groups remained significantly lower as compared to TGF-β3 stimulation (EdU/Ki-67 label for BMSC: *p* < 0.01, for ASC: *p* < 0.05). Although there were no significant differences between ASC and BMSC in controls or treatment groups, ASC showed a tendency for higher proliferation activity than BMSC.

**Figure 4:**
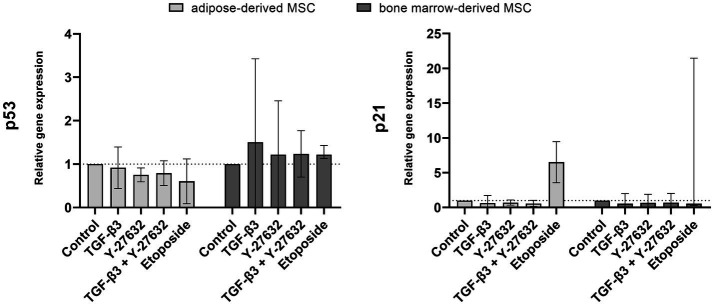
Gene expression of p53 and p21. ASC and BMSC were treated with 10 ng/mL TGF-β3, 10 μM Y-27632, in combination or 10 μM etoposide and analyzed after 48 h incubation. Bars represent the median values, error bars the 95% confidence intervals.

Gene expression of p21 and p53 were examined to assess senescence induction (Figure 4). Stimulation with TGF-β3, Y-27632, or in combination did not significantly alter the expression of p21 in ASC or BMSC. The addition of etoposide resulted in an upregulation of p21 expression in ASCs but was variable between BMSC donor horses with only one donor responding with an upregulation. Similarly, no significant regulation by TGF-β3, Y-27632, or in combination was detected for p53 gene expression.

## Discussion

Adipose tissue and bone marrow represent important sources for obtaining MSC for therapeutic purposes. Several studies show that ASC and BMSC are comparable in many biological responses. Nevertheless, differences between the two cell types have been reported in several studies where comparisons have been made. In this study, while cell morphology was found to be similar between the two cell types, BMSC tended to show lower proliferation activity than ASC. These results are consistent with other studies, where the MSC showed the same cell morphology as ASC but the doubling time of ASC was significantly higher than that of BMSC (9, 31, 32). Y-27632 induced an enlarged, less spindle-shaped cell shape which may be a result of cytoskeletal changes from inhibition of the Rho/ROCK pathway (33, 34). The effect on morphology is reversible (35) although this was not investigated in this study.

TGF-β3 is a well-established tool for initiating and supporting MSC differentiation. The Rho/ROCK inhibitor Y-27632 has been reported to have beneficial effects, particularly on cell proliferation and senescence, in the culture of embryonic stem cells and multipotent cells. Based on this, combined stimulation with Y-27632 and TGF-β3 was used to investigate potential synergistic effects as previously reported with respect to differentiation (17, 36). In our study, no significant effects on proliferation or senescence markers were found. This is in contrast to studies describing an increase of proliferation in human urine-derived mesenchymal stromal cells, periodontal ligament stem cells and other mesenchymal progenitor cells (19, 20, 37, 38), or a decrease in human adipose-derived MSC and CD34+ hematopoietic progenitor cells (28, 39) with Y-27632 stimulation. These varied responses suggest that Y-27632 likely targets multiple cellular pathways depending on the tissue origin of the cells.

Differences in senescence have also been reported for Y-27632-stimulated cells. Studies on primary keratinocytes showed a protective effect of Y-27632 that prevented cell senescence (16, 19), whereas it drove senescence in primary fibroblasts (40). In the current study, no significant regulation of senescence by Y-27632 was noted in either cell type with respect to p21 and p53 expression. It is possible that the age of the cells, in terms of passage number and population doubling level, and the different analyses used to investigate senescence between studies could play a role in varied cellular responses. The cells used in this study were at low passage number where they are at a young cellular age and likely to be more resilient to be driven towards a senescence phenotype and thus the potential protective effect of Y-27632 may not be readily apparent. It is possible that equine ASC and BMSC with higher cellular age (passage number > 15) may be more responsive to the effects of inhibiting the Rho/ROCK pathway. Further analysis would be interesting at late passage to rule out the senescence, as senescence is not be mediated solely by the p53/p21 signaling pathway.

A limitation of the study is the timepoint of investigations. This study focused on the initial effect on proliferation activity and senescence by Y-27632 and TGF-β3 stimulation at 48 h. Further experiments are necessary to investigate effects at longer time points.

## Conclusion

The Rho/ROCK inhibitor Y-27632 has no significant effect on proliferation and does not induce senescence in equine ASC and BMSC. Consequently, the inhibitor does not appear to be suitable as a proliferation-promoting supplement for cell cultivation. Nevertheless, no adverse effects are expected for short-term use for differentiation of equine MSC.

## Data availability statement

The original contributions presented in the study are included in the article/supplementary material, further inquiries can be directed to the corresponding author.

## Author contributions

MM created the study design, conducted the study, performed the statistical analysis, and wrote the first draft of the manuscript. JB and JD contributed to the conception and design of the study. JD wrote a section (parts of the methods and discussion) of the manuscript. DG provided the characterization data and prepared the related part of the manuscript. All authors contributed to the article and approved the submitted version.

## Funding

This work was supported by a fellowship from the International Giessen Graduate Centre for the Life Sciences.

## Conflict of interest

The authors declare that the research was conducted in the absence of any commercial or financial relationships that could be construed as a potential conflict of interest.

## Publisher’s note

All claims expressed in this article are solely those of the authors and do not necessarily represent those of their affiliated organizations, or those of the publisher, the editors and the reviewers. Any product that may be evaluated in this article, or claim that may be made by its manufacturer, is not guaranteed or endorsed by the publisher.

## References

[1] Prządka P Buczak K Frejlich E Gąsior L Suliga K Kiełbowicz Z. The role of mesenchymal stem cells (MSCs) in veterinary medicine and their use in musculoskeletal disorders. Biomolecules. (2021) 11:1141. doi: 10.3390/biom11081141, PMID: 34439807 PMC8391453

[2] Arnhold SJ Goletz I Klein H Stumpf G Beluche LA Rohde C. Isolation and characterization of bone marrow-derived equine mesenchymal stem cells. Am J Vet Res. (2007) 68:1095–105. doi: 10.2460/ajvr.68.10.1095, PMID: 17916017

[3] Koch TG Heerkens T Thomsen PD Betts DH. Isolation of mesenchymal stem cells from equine umbilical cord blood. BMC Biotechnol. (2007) 7:26. doi: 10.1186/1472-6750-7-26, PMID: 17537254 PMC1904213

[4] de Mattos Carvalho A Alves ALG Golim MA Moroz A Hussni CA de Oliveira PGG. Isolation and immunophenotypic characterization of mesenchymal stem cells derived from equine species adipose tissue. Vet Immunol Immunopathol. (2009) 132:303–6. doi: 10.1016/j.vetimm.2009.06.014, PMID: 19647331

[5] Ribitsch I Burk J Delling U Geißler C Gittel C Jülke H. Basic science and clinical application of stem cells in veterinary medicine. Adv Biochem Eng Biotechnol. (2010) 123:219–63. doi: 10.1007/10_2010_66 20309674

[6] Spaas JH de Schauwer C Cornillie P Meyer E van Soom A van de Walle GR. Culture and characterisation of equine peripheral blood mesenchymal stromal cells. Vet J. (2013) 195:107–13. doi: 10.1016/j.tvjl.2012.05.006, PMID: 22717781

[7] Pittenger MF Mackay AM Beck SC Jaiswal RK Douglas R Mosca JD. Multilineage potential of adult human mesenchymal stem cells. Science. (1999) 284:143–7. doi: 10.1126/science.284.5411.143 10102814

[8] Kargozar S Mozafari M Hashemian SJ Brouki Milan P Hamzehlou S Soleimani M. Osteogenic potential of stem cells-seeded bioactive nanocomposite scaffolds: a comparative study between human mesenchymal stem cells derived from bone, umbilical cord Wharton’s jelly, and adipose tissue. J Biomed Mater Res B Appl Biomater. (2018) 106:61–72. doi: 10.1002/jbm.b.33814, PMID: 27862947

[9] Mohamed-Ahmed S Fristad I Lie SA Suliman S Mustafa K Vindenes H. Adipose-derived and bone marrow mesenchymal stem cells: a donor-matched comparison. Stem Cell Res Ther. (2018) 9:168. doi: 10.1186/s13287-018-0914-1, PMID: 29921311 PMC6008936

[10] Wu W Le AV Mendez JJ Chang J Niklason LE Steinbacher DM. Osteogenic performance of donor-matched human adipose and bone marrow mesenchymal cells under dynamic culture. Tissue Eng Part A. (2015) 21:1621–32. doi: 10.1089/ten.TEA.2014.0115, PMID: 25668104 PMC4426327

[11] Riento K Ridley AJ. Rocks: multifunctional kinases in cell behaviour. Nat Rev Mol Cell Biol. (2003) 4:446–56. doi: 10.1038/nrm1128, PMID: 12778124

[12] Kang PH Schaffer DV Kumar S. Angiomotin links ROCK and YAP signaling in mechanosensitive differentiation of neural stem cells. Mol Biol Cell. (2020) 31:386–96. doi: 10.1091/mbc.E19-11-0602, PMID: 31940260 PMC7183791

[13] Li W Zhao J Wang J Sun L Xu H Sun W. ROCK-TAZ signaling axis regulates mechanical tension-induced osteogenic differentiation of rat cranial sagittal suture mesenchymal stem cells. J Cell Physiol. (2020) 235:5972–84. doi: 10.1002/jcp.29522, PMID: 31970784

[14] Maharam E Yaport M Villanueva NL Akinyibi T Laudier D He Z. Rho/ROCK signal transduction pathway is required for MSC tenogenic differentiation. Bone Res. (2015) 3:15015. doi: 10.1038/boneres.2015.15, PMID: 26509098 PMC4605238

[15] Kamishibahara Y Kawaguchi H Shimizu N. Rho kinase inhibitor Y-27632 promotes neuronal differentiation in mouse embryonic stem cells via phosphatidylinositol 3-kinase. Neurosci Lett. (2016) 615:44–9. doi: 10.1016/j.neulet.2016.01.022, PMID: 26797580

[16] Li Z Han S Wang X Han F Zhu X Zheng Z. Rho kinase inhibitor Y-27632 promotes the differentiation of human bone marrow mesenchymal stem cells into keratinocyte-like cells in xeno-free conditioned medium. Stem Cell Res Ther. (2015) 6:17. doi: 10.1186/s13287-015-0008-2, PMID: 25889377 PMC4393638

[17] Melzer M Schubert S Müller SF Geyer J Hagen A Niebert S. Rho/ROCK inhibition promotes TGF-β3-induced tenogenic differentiation in mesenchymal stromal cells. Stem Cells Int. (2021) 2021:8284690. doi: 10.1155/2021/8284690, PMID: 34659420 PMC8519677

[18] Kümper S Mardakheh FK McCarthy A Yeo M Stamp GW Paul A. Rho-associated kinase (ROCK) function is essential for cell cycle progression, senescence and tumorigenesis. elife. (2016) 5:e12994. doi: 10.7554/eLife.12203 PMC4798951 26765561

[19] Chapman S McDermott DH Shen K Jang MK McBride AA. The effect of rho kinase inhibition on long-term keratinocyte proliferation is rapid and conditional. Stem Cell Res Ther. (2014) 5:60. doi: 10.1186/scrt449, PMID: 24774536 PMC4055106

[20] Joo HJ Choi D-K Lim JS Park J-S Lee S-H Song S. ROCK suppression promotes differentiation and expansion of endothelial cells from embryonic stem cell-derived Flk1(+) mesodermal precursor cells. Blood. (2012) 120:2733–44. doi: 10.1182/blood-2012-04-421610, PMID: 22896004

[21] Jung B Lee H Kim S Tchah H Hwang C. Effect of rho-associated kinase inhibitor and mesenchymal stem cell-derived conditioned medium on corneal endothelial cell senescence and proliferation. Cells. (2021) 10:1463. doi: 10.3390/cells10061463, PMID: 34207965 PMC8230597

[22] Claassen DA Desler MM Rizzino A. ROCK inhibition enhances the recovery and growth of cryopreserved human embryonic stem cells and human induced pluripotent stem cells. Mol Reprod Dev. (2009) 76:722–32. doi: 10.1002/mrd.21021, PMID: 19235204 PMC3257892

[23] Martin-Ibañez R Unger C Strömberg A Baker D Canals JM Hovatta O. Novel cryopreservation method for dissociated human embryonic stem cells in the presence of a ROCK inhibitor. Hum Reprod. (2008) 23:2744–54. doi: 10.1093/humrep/den316, PMID: 18716037

[24] Godwin EE Young NJ Dudhia J Beamish IC Smith RKW. Implantation of bone marrow-derived mesenchymal stem cells demonstrates improved outcome in horses with overstrain injury of the superficial digital flexor tendon. Equine Vet J. (2012) 44:25–32. doi: 10.1111/j.2042-3306.2011.00363.x, PMID: 21615465

[25] Guest DJ Dudhia J Smith RKW Roberts SJ Conzemius M Innes JF. Position statement: minimal criteria for reporting veterinary and animal medicine research for mesenchymal stromal/stem cells in orthopedic applications. Front Vet Sci. (2022) 9:817041. doi: 10.3389/fvets.2022.817041, PMID: 35321059 PMC8936138

[26] Guest DJ Ousey JC Smith MR. Defining the expression of marker genes in equine mesenchymal stromal cells. Stem Cells Cloning. (2008) 1:1–9. doi: 10.2147/sccaa.s3824, PMID: 24198500 PMC3781685

[27] Yang H Wang H Ren J Chen Q Chen ZJ. cGAS is essential for cellular senescence. Proc Natl Acad Sci U S A. (2017) 114:E4612–20. doi: 10.1073/pnas.1705499114, PMID: 28533362 PMC5468617

[28] Lamas NJ Serra SC Salgado AJ Sousa N. Failure of Y-27632 to improve the culture of adult human adipose-derived stem cells. Stem Cells Cloning. (2015) 8:15–26. doi: 10.2147/SCCAA.S66597, PMID: 25609984 PMC4293935

[29] Hernandez-Segura A Nehme J Demaria M. Hallmarks of cellular senescence. Trends Cell Biol. (2018) 28:436–53. doi: 10.1016/j.tcb.2018.02.001 29477613

[30] Pfaffl MW. A new mathematical model for relative quantification in real-time RT-PCR. Nucleic Acids Res. (2001) 29:e45:e45e. doi: 10.1093/nar/29.9.e45, PMID: 11328886 PMC55695

[31] Li X Bai J Ji X Li R Xuan Y Wang Y. Comprehensive characterization of four different populations of human mesenchymal stem cells as regards their immune properties, proliferation and differentiation. Int J Mol Med. (2014) 34:695–704. doi: 10.3892/ijmm.2014.1821, PMID: 24970492 PMC4121354

[32] de Ugarte DA Morizono K Elbarbary A Alfonso Z Zuk PA Zhu M. Comparison of multi-lineage cells from human adipose tissue and bone marrow. Cells Tissues Organs. (2003) 174:101–9. doi: 10.1159/000071150, PMID: 12835573

[33] Ishizaki T Uehata M Tamechika I Keel J Nonomura K Maekawa M. Pharmacological properties of Y-27632, a specific inhibitor of rho-associated kinases. Mol Pharmacol. (2000) 57:976–83. PMID: 10779382

[34] Xu B Song G Ju Y Li X Song Y Watanabe S. RhoA/ROCK, cytoskeletal dynamics, and focal adhesion kinase are required for mechanical stretch-induced tenogenic differentiation of human mesenchymal stem cells. J Cell Physiol. (2012) 227:2722–9. doi: 10.1002/jcp.23016, PMID: 21898412

[35] Rao PV Deng PF Kumar J Epstein DL. Modulation of aqueous humor outflow facility by the rho kinase-specific inhibitor Y-27632. Invest Ophthalmol Vis Sci. (2001) 42:1029–37. PMID: 11274082

[36] Ji H Tang H Lin H Mao J Gao L Liu J. Rho/Rock cross-talks with transforming growth factor-β/Smad pathway participates in lung fibroblast-myofibroblast differentiation. Biomed Rep. (2014) 2:787–92. doi: 10.3892/br.2014.323, PMID: 25279146 PMC4179758

[37] Kim K Gil M Dayem AA Choi S Kang G-H Yang G-M. Improved isolation and culture of urine-derived stem cells (USCs) and enhanced production of immune cells from the USC-derived induced pluripotent stem cells. J Clin Med. (2020) 9:827. doi: 10.3390/jcm9030827, PMID: 32197458 PMC7141314

[38] Wang T Kang W Du L Ge S. Rho-kinase inhibitor Y-27632 facilitates the proliferation, migration and pluripotency of human periodontal ligament stem cells. J Cell Mol Med. (2017) 21:3100–12. doi: 10.1111/jcmm.13222, PMID: 28661039 PMC5661246

[39] Bueno C Montes R Menendez P. The ROCK inhibitor Y-27632 negatively affects the expansion/survival of both fresh and cryopreserved cord blood-derived CD34+ hematopoietic progenitor cells: Y-27632 negatively affects the expansion/survival of CD34+HSPCs. Stem Cell Rev Rep. (2010) 6:215–23. doi: 10.1007/s12015-010-9118-5, PMID: 20180051

[40] Li X Zhou Q Wang S Wang P Li J Xie Z. Prolonged treatment with Y-27632 promotes the senescence of primary human dermal fibroblasts by increasing the expression of IGFBP-5 and transforming them into a CAF-like phenotype. Aging. (2020) 12:16621–46. doi: 10.18632/aging.103910, PMID: 32843583 PMC7485707

